# Environmental Foundations of Typhoid Fever in the Fijian Residential Setting

**DOI:** 10.3390/ijerph16132407

**Published:** 2019-07-06

**Authors:** Aaron P. Jenkins, Stacy D. Jupiter, Adam Jenney, Varanisese Rosa, Alanieta Naucukidi, Namrata Prasad, Gandercillar Vosaki, Kim Mulholland, Richard Strugnell, Mike Kama, John A. Crump, Pierre Horwitz

**Affiliations:** 1 Centre for Ecosystem Management, Edith Cowan University, Joondalup, WA 6027, Australia; 2 School of Public Health, University of Sydney, Sydney, NSW 2006, Australia; 3 Wildlife Conservation Society, Suva, Fiji; 4 College of Medicine, Nursing and Health Sciences, Fiji National University, Suva, Fiji; 5 New Vaccines Group, Murdoch Children’s Research Institute, Parkville, VIC 3052, Australia; 6 Department of Preventative and Social Medicine, University of Otago, Dunedin 9054, New Zealand; 7 Department of Infectious Disease Epidemiology, London School of Hygiene and Tropical Medicine, London WC1H9SH, UK; 8 Department of Microbiology and Immunology, Peter Doherty Institute for Infection and Immunity, University of Melbourne, Melbourne, VIC 3000, Australia; 9 Fiji Centre for Communicable Disease Control, Fiji Ministry of Health and Medical Services, Suva, Fiji

**Keywords:** typhoid fever, drainage, residential setting, Fiji, water and soil

## Abstract

Proximal characteristics and conditions in the residential setting deserve greater attention for their potential to influence typhoid transmission. Using a case-control design in Central Division, Republic of Fiji, we examined bacterial (coliform and *Escherichia coli*) contamination and chemical composition of water and soil as potential vehicles of exposure to *Salmonella* Typhi, combining observational analysis of residential living conditions, geospatial analysis of household locations, and factor analysis to explore multivariate associations with the risk of developing typhoid fever. Factors positively associated with typhoid infection related to drainage [phosphate (OR 4.235, *p* = 0.042) and *E. coli* concentrations (OR 2.248, *p* = 0.029) in toilet drainage soil, housing [external condition (OR 3.712, *p* < 0.001)], drinking water contamination (OR 2.732, *p* = 0.003) and sanitary condition (OR 1.973, *p* = 0.031). These five factors explained 42.5% of the cumulative variance and were significant in predicting typhoid infection. Our results support the hypothesis that a combination of spatial and biophysical attributes of the residential setting influence the probability of typhoid transmission; in this study, factors associated with poor drainage, flooding, and sanitary condition increase local exposure to contaminated water and soil, and thereby infection. These findings extend testing of causal assumptions beyond the immediate domestic domain, enhance the scope of traditional case control epidemiology and allow greater specificity of interventions at the scale of the residential setting.

## 1. Introduction

Modelling differential risk of typhoid for use in policymaking and intervention requires defining local parameters to determine the relative importance of short-cycle (household) and long-cycle (environmental) transmission and the contributions of acute shedding, and convalescent and chronic carriage within the residential setting [1]. While behaviours associated with faecal contamination of food and water have dominated perspectives on typhoid transmission [2], determinants related to the residential setting, namely the conditions of the lived environment including infrastructure, and microbiological and physicochemical characteristics, warrant attention for their potential to influence risk of transmission. The classic case-control study remains the most widely used epidemiological approach for assessing risk of transmissible diseases such as typhoid fever, and for testing causal hypotheses in the proximal environment [3], although recent geospatial studies have also shed light on risk factors at broader spatial scales [4,5,6,7].

Outside of faecal contamination of drinking water, a statistically high-incidence of typhoid has been associated with local climate, elevation, and proximity to altered land and hydrologic systems [4,5,6,8,9,10]. In addition, household features have been frequently associated with increased risk, including the use of untreated surface waters (e.g., rivers, streams, wells) as drinking water [11,12]; poor water storage practices [13,14]; the use of contaminated bathing water [15]; the condition of the toilet or latrine [16,17]; and crowding of people and houses [18,19]. Inadequate drainage around the house and community has been significantly associated with increased risk of several enteric and diarrhoeal diseases [20,21,22]. Furthermore, the microbiological and biochemical properties of environmental reservoirs can also be associated with typhoid fever. A study in Kathmandu, Nepal, revealed that thermotolerant coliforms, nitrates, nitrites, turbidity, and ammonia in drinking water were positively correlated with the presence of *Salmonella* Typhi and *Salmonella* Paratyphi A nucleic acids, suggesting that chemical pollution of water in that setting was likely driven by rainfall runoff and localised contamination with human faecal waste [23]. From these studies, we propose that the characteristics and condition of the residential setting contain important determinants of typhoid fever transmission.

We used a case-control design to identify environmental risk factors operating at a residential level in Fiji associated with typhoid fever transmission, by increasing local exposure to faecally contaminated water and soil. We specifically investigated bacterial contamination and chemical composition of water and soil as vehicles of exposure and complemented these data with an observational analysis of residential living conditions and a quantitative spatial analysis of household position to assess risk and provide direction towards identifying intervention strategies. This combined approach not only describes the condition of the environment into which *Salmonella* Typhi is shed, but also extends the testing of causal assumptions beyond the immediate domestic domain.

## 2. Materials and Methods

### 2.1. Study Setting

#### 2.1.1. Geography and Demography

The Republic of Fiji (12–22° S and 176° E–178° W) has a total land area of 18,270 km^2^ spread across an archipelago of 332 islands [24]. Our study was confined to the wettest and most populous southeastern half of the largest island of Viti Levu (10,642 km^2^) in Central Division (4293 km^2^), one of Fiji’s four Divisions. This most populated area of Fiji (370,570 people) contains five provinces, includes the capital city of Suva (174,000 people), and is inhabited by 56.8% indigenous Fijians (*iTaukei*), 37.5% Fijians of Indian descent, and 5.7% of other ethnicities. Much of Central Division population resides in Suva with the remainder in small rural villages and settlements proximal to major watercourses. The southeastern half of Viti Levu has a mean annual rainfall of >3200 mm, concentrated during the cyclone season (November to May) [25]. The island of Viti Levu has steep slopes, large rivers and well- developed estuaries along coastal floodplains and complex geological origins [24].

#### 2.1.2. Typhoid Epidemiology

Typhoid in Fiji is endemic with incidence increasing since the 1990s [26], rising rapidly after 2004–2005, and exceeding a crude annual incidence of 52 cases per 100,000 in 2010 [27]. This precipitous rise in incidence may be explained by better surveillance and diagnostics, improved clinician awareness, and/or an actual increase in typhoid fever illnesses [28]. Since 2005, at least 18 typhoid outbreaks have been reported in Fiji [28]. In Fiji, young adults from 15 to 30 years of age present with acute infection most frequently, in contrast to many other typhoid endemic areas where children under five years of age are the peak age for diagnosis of with illness [28]. Ninety percent of reported Fijian cases are among *iTaukei* (Indigenous Fijians) [29]. These demographics may be misleading, as private health care data are unavailable and blood is rarely cultured from young children. In addition, access to healthcare from the two major ethnic groups may vary [30]. Case numbers typically peak in January to June each year, lagging the timing of the rainy season (November to April) by two months [27,31], and outbreaks have been reported following cyclones and flooding [32]. While the total number of cases is highest in urban areas, surveillance data and recent geospatial studies suggest typhoid is becoming increasingly common in rural areas [7,28].

#### 2.1.3. Access to Safe Water and Sanitation

Little progress has been made in the past two decades to improve access to microbiologically safe water and adequate sanitation in the Oceania region, where two-thirds of the population rely on unprotected drinking water sources and unsanitary means of excreta disposal, posing serious risks to health [33]. While published statistics for Fiji show 96% access to improved drinking water and 91% access to improved sanitation [33], these data do not indicate safety from microbial pathogens. An “improved” drinking water facility is generally one that “adequately protects the water from outside contamination” and includes piped household connections [34]. While municipal water is largely treated, many rural and peri-urban households have piped household connections into the house or yard coming from inadequately protected and untreated surface sources [12], which are unaccounted for by this definition. “Improved” sanitation “hygienically separates human excreta from human contact” including septic systems, pour flush and improved pit latrines [33]. The most recent Fiji government estimates were that 23% of the population was connected to municipal sewerage, 40% to septic tanks, and 37% disposed their sewerage directly into land and marine environments [35]. Pour flush and improved pit latrines are very common in rural and peri-urban areas of Fiji but often shallow, subject to flooding, and built into permeable soil. Septic tanks are infrequently maintained and often undercut by erosion, leading to cracking and leakage into the environment [7]. For both scenarios of water and sanitation, Fiji is frequently failing to meet UN Sustainable Development Goal targets for drinking water (Target 6.1) and sanitation and hygiene (Target 6.2) [36].

### 2.2. Residential Setting Selection and Evaluation

#### 2.2.1. Selection of Residences

Residential settings were measured from participants enrolled in an ongoing neighbourhood, ethnicity, and age interval (<4 years, 5–14 years, 15–24 years, 25–34 years, 35–44 years, 45–54 years, 55–64 years, 65–74 years, >75 years) matched case-control study [12]. Patients seeking care at any of the health facilities in Central Division, who resided in Central Division, presented with a history of fever, had *Salmonella* Typhi isolated from blood culture at the Colonial War Memorial Hospital (CWMH) Clinical Microbiology Laboratory from 27 January 2014 to 30 July 2015 and whose consent/assent were obtained were defined as typhoid fever cases. Cases above the age of 18 years were eligible for enrolment from 27 January 2014 to 1 May 2014, thereafter all age groups were enrolled. Controls were people who matched the case in ethnicity, were within the same age interval and did not experience fever within the past one-month. To recruit controls, we spun a pen at the case residence and selected the nearest house 100 steps away from the pen and in the direction of its tip (control I). Following the pen tip direction, a second control was selected from a neighbouring village, preferably in the adjacent river basin in rural areas, or in the adjacent nursing zone for urban and peri- urban scenarios (control II). The process of pen spinning was repeated until two eligible controls for each case were identified. Eighty cases and 160 controls were enrolled by 30 July 2015.

Given the average two-week incubation period for *Salmonella* Typhi in immunologically naïve individuals, we located and obtained accurate geospatial data for all case and control places of usual residence during the two-week window prior to onset of fever, assuming this as the most probable location of the patient coming into contact with the pathogen. All enrolled cases and controls were contacted and interviewed about their place of residence during this two-week window and geo-located by taking the position with a Garmin Map 78sc handheld Global Positioning System (GPS) placed one metre from their front door. Living conditions, microbiological contamination and physicochemical qualities of routinely contacted water and soil were assessed in a subset of 126 of the enrolled residential settings (42 cases, 84 controls). The survey methodology for each type of sampling is detailed below in Section 2.2.3 and Section 2.3.

#### 2.2.2. Geographical Position

Geospatial data layers were used as inputs for deriving potential spatial risk factors for typhoid at a residential level. Table 1 describes the layers, their sources, and the basic processing performed before potential spatial risk factors were derived. Using ArcMap 10.2 (Environmental Systems Research Institute, Redlands, CA, USA), elevation, and slope were precisely measured at the case and control geolocated household point and straight-line distance measurements were taken from this point to the nearest water body, nearest road, and nearest dense forest, as described in Jenkins et al. (2016) [7].

#### 2.2.3. Living Condition Evaluation

In association with collection of water and soil samples from study households, photographs were taken of each house, the immediate external property surrounding the house, toilet and toilet drainage, bathing facilities, food gardens, and nearest water body to the household. For each household, notes were taken based on observation in relation to storm water drainage, substrate, house and yard condition, drinking water and bathing environs, solid waste disposal, condition of excreta disposal facilities, position of household garden relative to excreta disposal facility drainage and the presence of fecal odours near to this facility. Using photographs and notes, a post hoc evaluation of living conditions of all households (*n* = 126) was conducted using the evaluation rubric shown in Appendix Table A1. A random sample of nine de-identified sets of residential setting photographs and notes were given to three raters to confidently test for a greater than 0.61 kappa (substantial agreement) from four raters across five categories (alpha = 0.05, power = 0.8). Fleiss’ Kappa statistic of inter-rater reliability was used to assess the reliability of the rating measures by determining the agreement between multiple raters [37].

### 2.3. Collection and Analysis of Water and Soil Samples

Three water and three soil samples were sought from each residence, wherever possible (Appendix Table A2). Using sterile techniques, 250 mL each of stored drinking water, the direct source of this water, and water from the nearest stream or river were collected. The direct source was defined as the site from which stored water was obtained. Using sterile stainless-steel trowels and measuring cylinders, 500 mL of surface soil (to 10 cm depth) was taken from 50 cm in front of the toilet, the drainage of this facility, and the food garden closest to the house. Toilet drainage samples were taken on the downhill side, one metre from the structure for external facilities and directly adjacent to where the facility drainage pipe enters the ground for municipally connected sewerage. For septic tanks, samples were taken downhill directly adjacent to the tank. To obtain measurements from these soil samples, they were saturated with distilled water (500 mL at room temperature) and gently mixed for 2 min, then poured through a sterile stainless-steel sieve (3 mm mesh) into 500 mL Pyrex sampling bottles. For all samples (water and water from soil), in situ measurements of pH, temperature, electrical conductivity (EC), and dissolved oxygen concentration (DO) were taken from each sample using a Thermo Scientific Orion Star A329 (pH/ISE/EC/DO) (ThermoFisher, Scientific, Waltham, Massachusetts, USA) portable multi-meter, noting smell and colour, and placed directly into a cooler at 1–4 °C then transported within six hours to the Fiji Centre for Communicable Disease Control water laboratory for processing. In the laboratory, 50 mL aliquots of each sample were used to assess coliform and *Escherichia coli* contamination, 10 mL aliquots of undiluted water samples were used to measure turbidity and remaining samples were filtered to 0.45 microns (Nalgene Polysolfone PCTE filter), with filtrate retained for same day colorimetric measurement of reactive phosphorus (orthophosphate), nitrate (NO_3_-N), and ammonia (NH_3_-N). Turbidity and colorimetric measurements were made with a Hach DR900 (Hach, Loveland, CO, USA) portable colorimeter.

To assess the microbiological quality of the water and soil specifically related to faecal contamination, we used the most probable number (MPN) method. The MPN method estimates the density of viable microorganisms in a test sample. It is based upon the application of the theory of probability to the numbers of observed positive growth responses to a standard dilution series of sample inocula placed in a set number of culture media tubes [38]. We used the 3-tube method of MPN. We inoculated 10 mL of sample into 10 mL of MacConkey broth, followed by 1 mL and then 0.1 mL of sample into 5 mL tubes of MacConkey broth. A total of 9 tubes per sample were used, 3 tubes with 10 mL MacConkey broth and 6 tubes with 5 mL MacConkey broth and, within each, an inverted Durham tube. MacConkey broth was used for the detection of coliform bacteria while the Durham tube was used for the detection of gas that is produced by the metabolic action of microorganisms. The inoculated broths were incubated at 37 °C for 48 h. After incubation, each tube was examined and those that were positive (production of acid and gas) were counted. Production of gas within the Durham tube indicated a positive reaction for gas production, while change in the colour of the MacConkey broth from the original purple to yellow indicated a positive reaction for acid production. Positives were noted as both a colour change as well as gas production. McCrady’s Table was used to calculate MPN total number of coliforms in the sample [38]. A loop of all positive samples was placed into tubes of 3 mL of peptone water and then placed into a water bath at 44.5 °C overnight. The following day we added 1–2 drops of Kovak’s indole reagent to each. A brick red or bright red ring on the surface of the peptone indicated positivity for *E. coli*. McCrady’s table was used to calculate MPN of *E. coli* in the sample.

### 2.4. Data Analysis

Preliminary assessments of data normality were performed using a Shapiro–Wilk test. Non-parametric tests were selected based on each distinct data type meeting specific test assumptions and predominance of test use in current literature. Initially nonparametric tests (Mann–Whitney U for continuous, Kruskal–Wallis H for categorical) were performed for the 26 residential setting variables (5 spatial, 11 living condition and 10 microbiological/physicochemical) to assess differences between case and control residences. To determine the strength of association between variables, a Spearman’s rank correlation coefficient (ρ) resemblance matrix was created and significance was determined at α = 0.05 and α= 0.01 (df = 25). To reduce data to a smaller set of summary variables and to explore underlining structure of multivariate relationships, data from 108 residences (36 cases and 72 controls) were assessed by Exploratory Factor Analysis (EFA) using Maximum Likelihood extraction [39]. The most complete biophysical datasets (source drinking water and toilet drainage soil) were used as proxies for water and soil as they were significantly correlated (*p* < 0.01) across multiple parameters. Missing values were imputed using the Expectation-Maximization algorithm [40]. We used Varimax orthogonal rotation with Kaiser normalisation to simplify the columns of the factor matrix so that factor extracts were clearly associated and separation among the variables was shown [40]. Logistic regression was run using only significant factors (*p* < 0.01) to obtain odds ratios of each factor and a logistic function. The function constant is the expected value of the log-odds of typhoid risk when all of the predictor variables equal zero. Linear regressions of factor loadings against variables within each factor were used to establish relative contributions of variables within each factor and to establish a variable-based risk probability function. All statistics were performed using IBM SPSS Statistics for Windows, Version 22.0 (IBM Corporation, Armonk, NY, USA).

### 2.5. Research Ethics

Ethics approvals were obtained from the Fiji National Health Research Committee (FNHRC# 201370), the Human Research Ethics Committee of Edith Cowan University (Proj # 10017), and the Human Ethics Committee of the University of Otago. A research permit was obtained from the Fiji Ministry of Education, National Heritage Culture and Arts (Ref: RA 02/14) and permission was sought from provincial administrators and village chiefs before village visits. Verbal and written details of the study were provided in Fijian and/or English according to the participants’ preferences, and written informed consent was obtained from all participants. All data were de-identified prior to analysis.

## 3. Results

### 3.1. Proximal Residential Setting

**Figure 1:**
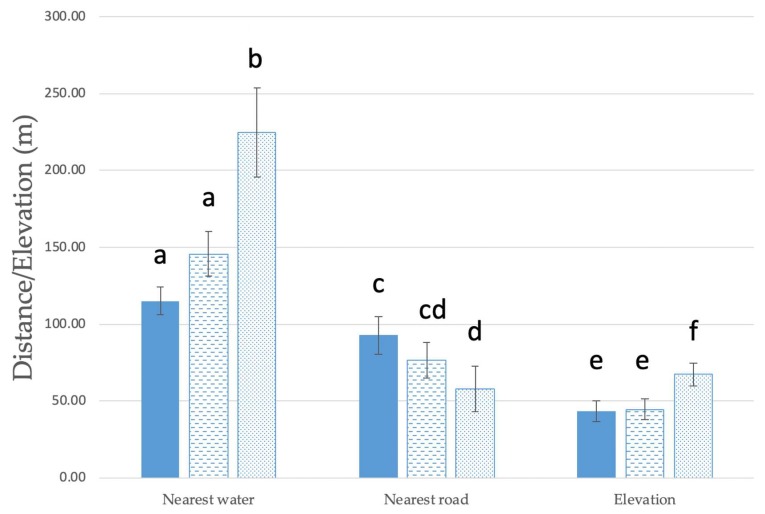
Mean proximal residential setting of typhoid cases versus control households in Central Division, Fiji (Cases; *n* = 80, Control I; *n* = 80, Control II; *n* = 80). Solid columns = Cases; dashed columns = Control I; dotted columns = Control II; error bars = +/− standard error. Only significant parameters are shown. Within each set of columns, sequential lettering indicates significant difference.

Spatial data revealed typhoid case residences to be significantly closer to flowing water bodies by an average of 110 m (Mann–Whitney U = 2537, *p* = 0.023 two tailed), further from the nearest road by an average of 35 m (Mann–Whitney U = 2359, *p* = 0.004 two tailed) and 24 m lower in elevation (Mann–Whitney U = 2713.5, *p* = 0.049 two tailed) on average than control II residences (Figure 1). We did not detect significant differences for these variables between case residences and control I residences.

### 3.2. Household Living Conditions

**Figure 2:**
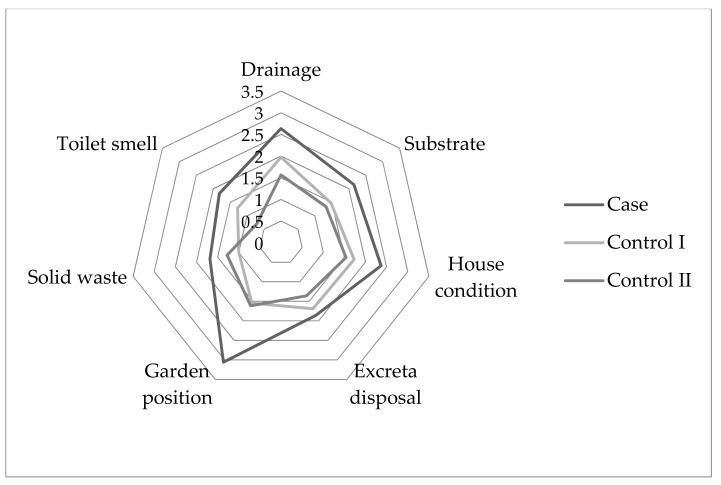
Mean rank of household living conditions for case versus controls in Central Division, Fiji based on a 0–4 rank of increased perceived likelihood of condition facilitating or indicating disease transmission. Only significant conditions are shown.

The Fleiss’ Kappa inter-rater reliability for post hoc evaluation of living conditions was found to be 0.62 (*p* < 0.05), indicating “substantial agreement” with the evaluation of the first author [36]. Several conditions in the lived environment of case households were significantly different from control households (Kruskal–Wallis H Test; *p* < 0.05) (Figure 2). In comparison to both controls, case residences had significantly poorer stormwater drainage (case vs. control I: χ^2^ = 8.758, *p* = 0.003; case vs. control II: χ^2^ = 18.993, *p* = 0.000); more exposed bare soil (case vs. control I: χ^2^ = 6.967, *p* = 0.008; case vs. control II: χ^2^ = 11.763, *p* = 0.001), poorer household condition (case vs. control I: χ^2^ = 5.543, *p* = 0.019; case vs. control II: χ^2^ = 10.063, *p* = 0.002) and food gardens nearby to toilet or septic drainage (case vs. control I: χ^2^ = 16.849, *p* = 0.000; case vs. control II: χ^2^ = 17.042, *p* = 0.000). Compared to control II residences, cases also had significantly less contained excreta disposal (i.e., damaged septic tank or pit latrine) (case vs. control II: χ^2^ = 4.330, *p* = 0.037) and greater smell of faeces near the toilet (case vs. control II: χ^2^ = 10.659, *p* = 0.001). Within the same community, case houses also had significantly higher amounts of unconstrained solid waste (case vs. control I: χ^2^ = 4.414, *p* = 0.036) nearby than control I houses.

### 3.3. Biophysical Parameters of Water and Soil (Summary Statistics in Appendix Table A3)

#### *Escherichia coli* in Stored Water

**Figure 3:**
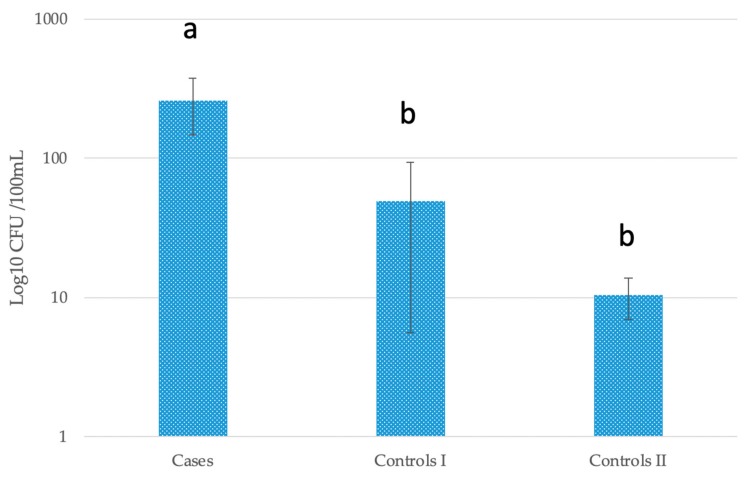
Mean most probable number (MPN) log 10 CFU of *E. coli* per 100 mL of drinking water stored by case and control households. Error bars = +/− standard error. Sequential lettering above columns indicates significant difference.

The concentration of *E. coli* in stored drinking water in case households was significantly higher than both control I (Mann–Whitney U = 316.5, *p* = 0.032 two tailed) and control II (Mann–Whitney U = 360.5, *p* = 0.023 two tailed) households, by factors of 5 and 25 respectively, whereas controls did not differ significantly from each other (Figure 3). Neither concentrations of *E. coli* nor coliforms in water sources or nearby streams were significantly different between case and control residences.

### 3.4. Physicochemical Parameters

**Figure 4:**
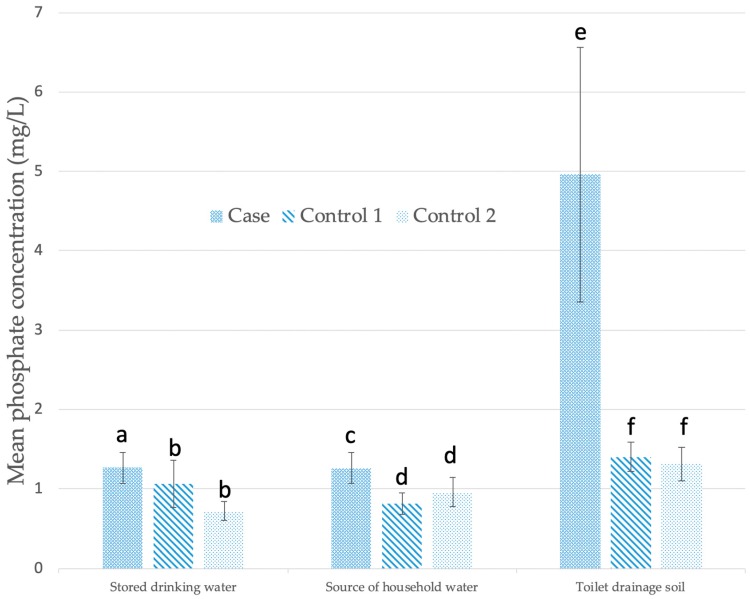
Mean concentration of phosphates in water and soil in the residential setting of cases and controls. error bars = +/−standard error. Within each set of columns, sequential lettering indicates significant difference.

The mean concentration of phosphate was significantly higher in stored drinking water (Mann–Whitney U = 294.5, *p* = 0.045 two tailed) and the drinking water source (Mann–Whitney U = 446.0, *p* = 0.023 two tailed) in case households compared to both control I and control II (Mann–Whitney U = 227.0, *p* = 0.007 two tailed; Mann–Whitney U = 508.5, *p* = 0.027 two tailed) households respectively, whereas controls did not differ significantly from each other. Cases households also had significantly higher phosphates in toilet drainage soil (Mann–Whitney U = 543.5, *p* = 0.03 two tailed) than control II households, whereas controls did not differ significantly from each other (Figure 4).

The mean concentration of ammonia was also significantly higher in stream water nearest to case households (Mann–Whitney U = 319.5, *p* = 0.03 two tailed) compared to control II households. Cases and control I stream water ammonia concentration did not differ significantly from each other, although control I and control II samples were significantly different (Mann–Whitney U = 251.0, *p* = 0.037 two tailed). Mean salinity (measured as EC) was also significantly higher in toilet drainage soil of cases than control II households (Mann–Whitney U = 535.0, *p* = 0.024 two tailed), whereas controls did not differ significantly from each other.

### 3.5. Factor Analysis

Initially, the factorability of 35 variables was examined and nearest water body, nearest forest and slope were excluded due to communalities below 0.3. All remaining communalities were above 0.3, confirming each variable shared some common variance with other items (Table 2); 30 of the 32 remaining variables were correlated at a level of 0.3 or higher with at least one other item. The Kaiser–Meyer–Olkin measure of sampling adequacy was 0.585, above a recommended value of 0.5 [39] and Bartlett’s test of sphericity was significant (χ^2^ = (496) = 1197.98, *p* < 0.05). Given these indicators, factor analysis was deemed suitable with 32 variables (Appendix Table A4). Exploratory factor analysis with Varimax rotation resulted in 11 factors based on eigenvalues greater than one, five of which were significant in predicting typhoid (78.7% correct) with a backwards Wald stepwise regression and explained 42.5% of the cumulative variance (Table 2). Variables clustered together along significant factors characterised by: [factor 1] external condition (related to substrate, drainage, household condition, amount of solid waste near house, and garden position); [factor 2] drinking water condition (related to *E. coli* concentration in source house water, drinking water storage, phosphate concentration in source house water, and distance to nearest road); [factor 3] sanitary condition (related to ammonia concentration in source house water and toilet smell); [factor 4] microbial loads (related to *E. coli* and ammonia concentration of toilet drainage soil (TDS)); and [factor 5] nutrient load (phosphate concentration) of TDS. The probable risk of typhoid exposure in this endemic Fijian residential setting can be expressed as the following logistic function:

e (−0.908 + 1.312 [External Condition] + 1.005 [Drinking Water Condition] +0.680 [Sanitary Condition] + 0.810 [TDS Microbial] + 1.443 [TDS Nutrient])(1)

The odds ratio of typhoid risk is highest for the factor associated with nutrient loading of toilet drainage soil (OR 4.235, *p* = 0.042), followed by factors loaded with variables associated with: external residential condition (OR 3.712, *p* = 0.000); drinking water condition (OR 2.732, *p* = 0.003); microbial contamination of toilet drain soil (OR 2.248, *p* = 0.029); and sanitary condition (OR 1.973, *p* = 0.031). Linear regression of risk factors against component variables resulted in the following five functions, which indicate relative importance of the variables within each risk factor:

1. External Condition = −1.830 + 0.384 (Substrate) + 0.249 (Drainage) + 0.197 (House condition) + 0.146 (Solid waste) +0.055 (Garden position)
2. Drinking Water Condition = −0.691 + 0.509 (Drinking water storage) + 0.201 (Phosphate SHW) + 0.002 (Nearest road) + 0.001 (*E. coli* SHW)
3. Sanitary Condition = −0.611 + 2.181 (Ammonia SHW) + 0.404 (Toilet smell)
4. Toilet Drainage−Microbial = −1.617 + 0.001 (*E. coli* TDS)
5. Toilet Drainage−Nutrient = −3.72 + 0.140 (Phosphate TDS)

## 4. Discussion

Our results support the hypothesis that multiple spatial and biophysical characteristics and conditions of the residential setting influence the probability of typhoid transmission. These appear to be associated with poor drainage, flooding and sanitation, which increase local exposure to contaminated water and soil. These effects are proximal, demonstrated by cases most commonly differing significantly from the more distant second control household. These particular observations and measurements can aid prediction of typhoid exposure risk in similar endemic settings and help to prioritise remedial measures.

### 4.1. External Conditions

There are several explanations for a strong relationship between typhoid exposure risk and the conditions found outside of the house in the residential setting. Poorly drained stormwater and household wastewater can create stagnant pools, providing sites for bacterial growth, exposure to pathogens, and breeding sites for several arthropod vectors [22]. Poor stormwater drainage can lead to flooding which may damage water supply or sanitation infrastructure. Further, where drainage and sanitation are inadequate, runoff can transport faeces across land and contaminate domestic water sources [41], household gardens and household wastewater may also contain pathogens that can pollute groundwater [42].

In our study, patients with typhoid fever residing at lower elevations and in closer proximity to surface water bodies (i.e., streams) had poorer drainage and significantly higher *E. coli* concentrations in stored drinking water. These findings are backed by previous studies that identified the importance of elevation in predicting typhoid risk across several spatial scales. For example, higher typhoid fever risk has been demonstrated with proximity to rivers [5,43], at low elevations [4,5,6], and in association with flooding [5,16]. Two related studies in Fiji also demonstrated increased typhoid incidence in low-lying areas where potential for flooding and exposure to contaminated runoff is higher [5,7]. While the proportion of exposed soil in the residential setting has yet to be reported as a risk factor for typhoid, the mechanisms by which exposed soil can facilitate increased microbial pathogen exposure are salient. Vegetated areas produce less runoff than bare soil or impervious surfaces [44]. Greater amounts of exposed soil in the residential setting contribute to higher local rates of erosion and runoff, and when combined with poor stormwater drainage, facilitate increased faecal and nutrient contamination of open water sources from runoff and a greater capacity to undermine sanitation infrastructure [7,41]. We found the relationship between proportion of bare soil and decreasing level of drainage to be significant (ρ = 0.563, *p* = 0.000), as runoff from bare ground contributes directly to drainage congestion, stagnant pool formation, water logging, and increased eutrophication [44]. The high water retention and bacterial adsorption properties of clay loams, the primary soil type in the Central Division [45], also increase the likelihood of bacterial survival and transport into the house or nearby drainage [46]. In early experiments in various soil types, *S.* Typhi survival was most prolonged in clay loam (greater than 120 days) [47]. Typhoid incidence and recurrence in Central Division strongly correlate with area of high erosion risk at a sub-catchment scale, indicating a mechanistic connection to exposed soil across landscapes [7].

Our study also pinpoints that the type and maintenance of sanitation infrastructure is associated with increased typhoid risk. These results reflect both socio-economic status and occupant efforts in household maintenance and waste disposal. The mechanisms by which housing conditions and solid waste can affect likelihood of typhoid occurrence have both direct and indirect biological (e.g., poor sanitary conditions) and psychological pathways (e.g., apathy) [48]. These results are supported by the findings that typhoid fever was associated with poor housing [15], and strongly associated with a quality of life factor that included mean house price and proportion of slum dwellings [19]. Future research should therefore focus on direct comparisons of socio-economic status and hygiene behaviours in assessing typhoid risk.

**Figure 5:**
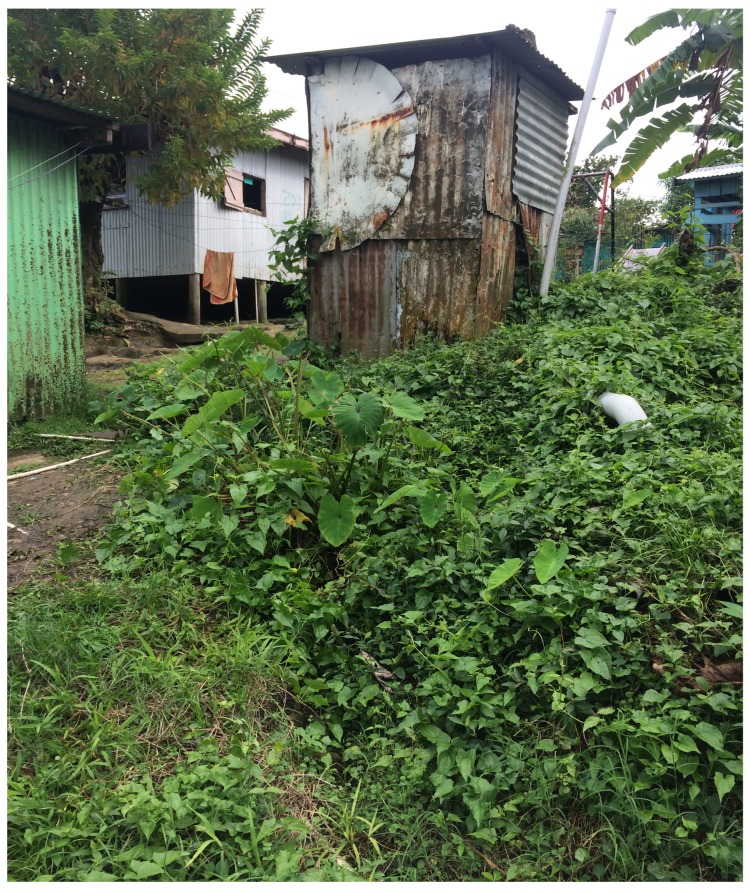
Household root crop gardens propagated directly on the toilet drainage area are common among Central Division typhoid cases (Photo credit: Aaron Jenkins).

In the Fijian context and throughout much of the Pacific, it is common practice to have a small garden of staple root crops (e.g., taro, cassava) near the house for domestic use. While propagating vegetables in nutrient-rich drainage areas is a common and traditional practice throughout the region [49], our study revealed that case household gardens were positioned significantly closer to the household toilet or septic tank, and the majority of cases (76%) propagated vegetables directly on or below the toilet drainage area (Figure 5). Garden position on or below the drainage area correlated significantly with ammonia in the garden soil and the smell of faeces near the toilet, suggesting faecal contamination. This vegetable propagation practice can be considered a form of passive use of human waste for fertiliser. While proximity of household garden to toilet drainage has never been specifically identified as a risk factor for typhoid, active fertilization of produce with human faeces has been implicated in long-cycle typhoid fever transmission [50]. Our recent case-control study in Fiji found that eating unwashed household garden produce was significantly associated with typhoid fever [12].

### 4.2. Drinking Water Conditions

We found that typhoid risk at the residential level is associated with household drinking water conditions. Mean concentrations of phosphates were significantly higher in both the stored drinking water and the source of drinking water in case households compared to controls. Phosphate readily binds to ultrafine (e.g., clay) sediment particles, which if washed into water sources can be a primary source of contamination [51]. This is noteworthy in light of findings that significant environmental determinants of typhoid at the sub-catchment level in Fiji are linked to increased risk of exposure from erosion prone areas [7]. Although sources of phosphates can be natural and anthropogenic, our finding that phosphate concentration in stored and source water is positively correlated with *E. coli* numbers and is significantly higher in the toilet drainage soil suggests faulty excreta disposal as a likely source. Among the thermotolerant coliforms, *E. coli* is the preferred microbial indicator of recent faecal contamination of drinking water and the possible presence of disease-causing pathogens [52]. While elevated phosphate in drinking water has not previously been reported as a risk factor for typhoid, a recent study revealed that thermotolerant coliforms, nitrates, nitrites, turbidity and ammonia in water were positively correlated with the presence of *Salmonella* Typhi and *Salmonella* Paratyphi A nucleic acids, suggesting that pollution of drinking water in this endemic setting is likely driven by localised contamination with human faecal waste [23]. While distal faecal contamination may also be occurring, the use of narrow-mouthed storage containers within the house may reduce contamination risks from unwashed hands dipping into stored water [14,53]. Again, our recent case-control study in Fiji found that frequent handwashing after defecating was independently associated with lower odds of typhoid fever [12].

Our univariate analysis showed significantly higher concentration of *E. coli* in stored drinking water in case households, although the source of this drinking water did not differ significantly, suggesting that contamination is occurring within the residential setting. The literature that deals with the relationship between *E. coli* contamination and typhoid risk is conflicted, with a study showing no difference in the microbiological water quality of home drinking water between cases and controls [53] where another found thermotolerant coliform numbers in source drinking water were positively correlated with the presence of *Salmonella* Typhi and *Salmonella* Paratyphi A nucleic acids [23]. It is noteworthy that the average concentration of *E. coli* in stored water across all our study households (cases and controls) was 115.24 CFU/100 mL (N = 83, Range 3–2400, SD 416.3), which is classified as “gross pollution” by WHO standards [52], indicating poor residential water quality in general. In addition, the concentration of *E. coli* in water of the nearest stream was substantially greater in case than control II households (Mann–Whitney U test; *p* = 0.059 two tailed), suggesting that variables that influence the external condition factor (e.g., poor drainage and exposed soil near the house) are likely both acting to enhance the risk of pathogen exposure during periods of heavy precipitation, either through secondary contamination of stored water or direct contamination of exposed water sources. While *E. coli* or thermotolerant coliforms in drinking water are important indicators of faecal contamination, they are imperfect and their presence does not necessarily equate with risk since water quality varies both temporally and spatially and occasional sampling may not accurately reflect actual pathogen exposure [52]. It has also been suggested that *E. coli* may be present, or even multiply, in tropical waters not subject to human faecal pollution [54], which could confound results. Cross tabulation or multivariate approaches combining results of sanitation surveys [52], and potential for flooding will likely yield enhanced predictive power.

In addition, typhoid case residences were significantly further from the nearest road compared to control II households (Figure 1). Rural residences in Fiji and the region (where roads are fewer) are typically more remote from municipal treated drinking water and sewerage services [35]. Roads also have drains and culverts, so residences closer to roads will have greater protection from surface water flows, and water will move more quickly through an area where drains and culverts are not blocked (with the reverse being true when they are not well maintained). While this variable has not been previously reported as a risk factor for typhoid, proximity to roads is a key factor for developing country communities adopting improved sanitation practices [55].

### 4.3. Sanitation Conditions

The exposure of individuals within a residential setting to improperly disposed excreta has been identified as a risk factor for typhoid in an endemic context [14], and is supported by our study findings that high nutrient and microbial concentration in toilet drainage soil and poor sanitary conditions are all associated with typhoid risk in Central Division, Fiji. For enteric diseases in general, it is suggested that prevention of excreta entering the domestic arena has a greater impact on health than behaviours preventing pathogens in the environment from being ingested (e.g., hand washing) [55]. A residential setting that has poor drainage and frequent flooding with unimproved pit latrines and damaged septic systems, situated in permeable, highly erodible soil, is highly conducive to typhoid transmission. Pit latrines have been shown to be a risk factor for typhoid in several studies both with and without flooding being implicated [12,16,17] and damaged sanitation infrastructure has been linked to several typhoid outbreaks [56].

### 4.4. Study Limitations

Controls may have been exposed to *S.* Typhi but, if afebrile over the last month, were eligible for recruitment. Many significant findings related to proximal residential position only relate to second controls external to the case community, highlighting the need for multiple controls in this type of study. Using observational and measured data as part of a case-control study design eliminates the often-criticized re-call bias [3], but can also introduce observational bias, as the observer is aware of which residences are cases and controls. We dealt with this for household living condition observations by measuring inter-rater reliability with de-identified observational data, however, this requires increased time and personnel investment. As single observations and measurements are made after the disease has occurred, one cannot ascribe causality to the factors that are measured or establish a timeline of exposure. For example, our measurements of high phosphates in the water and soil of cases could be explained by residual detergents remaining after cases attempt to clean up in anticipation of the study team arriving. While triangulation with other observational and measured variables suggests this is probably not the case, the alteration of behaviour by study subjects due to their awareness of being observed (Hawthorne effect) cannot be ruled out as a possibility. Seasonal variation may also introduce a level of variability that is unaccounted for in this design, resulting in elevated nutrient and microbial concentration after periods of heavy rainfall. As only 42.5% of the variance in typhoid risk was explained by residential setting factors, residual variance may be explained by factors operating at a larger scale or individual behaviours [7,12]. One of the commonly cited advantages of the case-control design is that it is relatively cheap and rapid [57] compared to cohort or randomised controlled trials, however, introducing intensive sampling, lab analysis and GIS into the design results in time constraints, increased associated costs and limits sample sizes.

## 5. Conclusions

While behavioural determinants, such as sanitary practice, are commonly recognised as important in the transmission of typhoid, environmental factors related to drainage, housing and the condition of water and sanitation provide the residential setting for these behaviours and therefore influence the risk of transmission. Our results objectively verify similar causal pathways for Fiji suggested by our recent interview-based case-control study, including unimproved sanitation and eating unwashed household garden produce. Environmental health practitioners can benefit from an interdisciplinary approach to categorizing the environment into which *Salmonella* Typhi is shed to extend the testing of causal assumptions beyond the immediate domestic domain, enhance the scope of traditional case-control epidemiological approaches and allow targeted water, sanitation and hygiene (WASH) interventions to be made with greater specificity at the residential level. In addition to improving sanitation facilities and protecting stored water and water sources from human feces, interventions in this residential setting should also include revegetation of exposed soil to reduce erosion and runoff, removing household gardens from toilet drainage areas and improving household stormwater and wastewater drainage.

## Figures and Tables

**Table 1:** Geospatial data layers, sources, and data processing, Fiji typhoid case-control study, 2014–2015.

Base Layer	Source	Dataset Details	Processing Details *
**Viti Levu coastline**	Fiji Department of Lands, National Government (NG)	NA	None
**Central Division boundary**	iTaukei Lands and Fisheries Commission, NG	NA	Removed small islands off Viti Levu
**Road network**	Fiji Roads Authority, NG	2015 update (sealed and unsealed)	None
**River network**	Fiji Department of Lands, NG	Primary network with 2nd order streams	None
**Creek network**	Fiji Department of Lands, NG	3rd order and higher creeks	Merged creek and river layers to create hydrology network layer
**Dense forest cover**	Fiji Department of Forestry (DoF), NG	Digitized from 2001 Landsat ETM+ data, verified against DoF vegetation maps of 2010	None
**Digital terrain model (DTM)**	Secretariat of the Pacific Community, Geoscience Division	25 m resolution with contour shading	None
**Typhoid case and control household positions**	This study	Digitized from GPS Map80 position, 1 m in front of house.	None

* All data transformed to UTM zone 60S with WGS 84 datum and processed in ArcMap 10.2 (ESRI).

**Table 2:** Summary of Exploratory Factor Analysis for residential risk of typhoid fever using Maximum Likelihood estimation with Varimax rotation showing communalities, % variance explained and eigenvalues (*n* = 497), Fiji typhoid case-control study, 2014–2015. Only significant factors and associated variables are shown. Factor loadings above 0.4 are shown in bold. SHW = source of house water; TDS = toilet drainage soil.

			FACTORS			
Variables	1 (External Condition)	2 (Drinking Water Condition)	3 (Sanitary Conditions)	4 (TDS Microbial)	5 (TDS Nutrient)	Communalities
Substrate	**0.827**	0.000	−0.008	0.035	−0.037	0.648
Drainage	**0.780**	0.177	0.157	−0.008	0.034	0.706
House condition	**0.724**	−0.003	0.006	−0.041	0.084	0.665
Solid Waste	**0.532**	−0.165	0.035	−0.185	−0.007	0.515
Garden position	**0.448**	0.279	0.236	−0.101	0.126	0.471
*E. coli* SHW	0.078	**0.665**	−0.138	−0.070	−0.110	0.487
Drinking water storage	0.007	**0.634**	0.151	0.090	0.170	0.506
Phosphate SHW	−0.049	**0.472**	−0.087	−0.145	0.047	0.408
Nearest road	0.055	**0.459**	0.149	−0.089	−0.028	0.427
Toilet smell	0.277	−0.054	**0.642**	0.008	0.045	0.510
Ammonia SHW	−0.009	0.094	**0.420**	0.165	−0.080	0.360
*E. coli* TDS	−0.032	−0.064	0.011	**0.592**	0.111	0.405
Ammonia TDS	−0.001	−0.128	0.356	**0.590**	0.085	0.368
Phosphate TDS	0.080	0.113	−0.077	0.171	**0.899**	0.452
Eigenvalue	4.193	3.010	2.516	1.976	1.892	
Cumulative % variance	13.1	22.5	30.4	36.5	42.5	

## Appendix A

**Table A1:** Rubric for evaluation of living conditions, Fiji typhoid case-control study, 2014–2015 *.

Category	Blank	0	1	2	3	4
**Bathing environs**	Do not know	Inside, piped, treated	Inside, piped, untreated	Outside, piped, treated	Outside, piped or unpiped, untreated	Outside, stream
**Drainage near house**	Do not know	Excellent	Good	Moderate	Minimal	Terrible
**Drinking water environs**	Do not know	Inside, piped, treated	Inside, piped, untreated	Outside, piped, treated	Outside, piped or unpiped, untreated	Outside, stream
**Drinking Water Storage**	Do not know/Not stored	Inside, closed mouth	Inside, open mouth	Outside, closed mouth	Outside, open mouth, sheltered	Outside, open mouth, unsheltered
**Faecal disposal**	Do not know	Flush to sewer line	Flush to intact septic	Flush to damaged septic	Improved Pit latrine	Unimproved pit latrine
**Garden position**	Do not know	Distant and above toilet or septic tank	Distant and level or below toilet or septic tank	Moderate distance from toilet or septic tank	Near and above toilet or septic	Directly below toilet or septic
**House condition**	Do not know	Well maintained	Few repairs needed	Moderate repairs needed	Large repairs needed	Major state of disrepair
**Housing density**	Do not know	Very distant	Distant	Moderately close	Very close	Against another house
**Smell near toilet**	Do not know	None	Slight smell	Moderate smell	Clear smell of faeces or rubbish	Very strong smell of faeces or rubbish
**Solid waste near house**	Do not know	None	Little	Moderate	High	Very High
**Substrate near house**	Do not know	Paved	Fully Vegetated	Moderately vegetated	Minimally vegetated	Bare soil

* Based on a 0–4 rank of increased perceived likelihood of parameter facilitating or indicating disease transmission. “Blank” was regarded as equivalent to a missing value.

Table A2 Sample sizes for microbiological/physicochemical parameters measured in residential (**a**) water and (**b**) soil (water from soil) samples, Fiji typhoid case-control study, 2014–2015. C = cases; CI = control I; CII = Control II; # = number.

**Table:** (**a**)

Microbiological/Physicochemical Parameter	Stored Drinking Water			Drinking Water Source			Nearest Stream Water		
	**C**	**CI**	**CII**	**C**	**CI**	**CII**	**C**	**CI**	**CII**
**Coliforms (CFU/100 mL)**	31	25	27	38	36	41	30	25	16
***E. coli* (CFU/100 mL)**	31	25	27	38	36	41	25	16	15
**Turbidity (FTU)**	31	22	27	33	32	35	20	13	9
**Phosphate (mg/L)**	30	24	26	37	35	39	27	13	14
**Nitrate (mg/L)**	30	24	26	36	35	39	25	15	14
**Ammonia (mg/L)**	30	24	26	37	36	40	27	17	17
**Electrical Conductivity (uS/cm)**	30	25	25	37	35	39	25	15	12
**Temperature (°C)**	30	25	25	37	35	39	25	14	12
**Dissolved Oxygen (mg/L)**	28	23	23	36	34	37	23	14	11
**pH**	30	25	25	37	35	39	24	15	12
**# Measurements taken**	301	242	257	366	349	389	251	157	132

**Table:** (**b**)

Microbiological/Physicochemical Parameter	Toilet Soil			Toilet Drainage Soil			Household Garden Soil		
	**C**	**CI**	**CII**	**C**	**CI**	**CII**	**C**	**CI**	**CII**
**Coliforms (CFU/100 mL)**	31	16	9	40	37	40	32	28	27
***E. coli* (CFU/100 mL)**	31	15	9	40	37	49	31	28	27
**Phosphate (mg/L)**	30	14	9	37	36	38	27	28	25
**Nitrate (mg/L)**	30	15	9	39	38	32	32	29	26
**Ammonia (mg/L)**	30	15	9	37	39	34	27	29	27
**Electrical Conductivity (uS/cm)**	30	14	9	40	36	38	33	28	25
**Temperature (°C)**	30	14	9	40	36	38	33	28	25
**Dissolved Oxygen (mg/L)**	28	13	9	38	34	36	32	28	24
**pH**	30	14	9	40	36	38	33	28	25
**# Measurements taken**	270	130	81	351	329	343	280	254	231

Table A3 Summary statistics for microbiological/physicochemical parameters measured in residential water (**a**) and (**b**) soil (water from soil) samples, Fiji typhoid case-control study, 2014–2015. *n* = sample size; $\bar{x}$ = mean; R = range; SD = Standard deviation.

**Table:** (**a**)

Microbiological/Physicochemical Parameter	Stored Drinking Water				Drinking Water Source				Nearest Stream Water			
	*n*	$\bar{x}$	R	SD	*n*	$\bar{x}$	R	SD	*n*	$\bar{x}$	R	SD
**Coliforms (CFU/100 mL)**	83	274	2397	639.3	115	181	2397	521.8	71	1495.1	2397	991.3
***E. coli* (CFU/100 mL)**	83	115.2	2397	416.6	115	55.8	1097	186.2	56	802.1	2397	940.2
**Turbidity (FTU)**	83	2.2	27	3.6	100	3.3	32	5.0	42	31.4	807	125.5
**Phosphate (mg/L)**	80	1.0	6.0	1.1	111	1.0	6.1	1.1	54	1.3	8.3	1.5
**Nitrate (mg/L)**	80	0.02	0.31	0.05	110	1.0	6.1	1.1	54	0.1	2.7	0.4
**Ammonia (mg/L)**	80	0.03	0.89	0.11	113	0.04	1.1	0.14	61	2.3	50	9.3
**Electrical Conductivity (uS/cm)**	80	454.9	2046.6	451.5	111	273.9	1943.1	360	52	218.5	1557.1	293.2
**Temperature (°C)**	80	25.9	18.3	3.1	111	26.4	10.8	2.3	51	25.8	12	2.3
**Dissolved Oxygen (mg/L)**	74	97.9	50.7	7.5	98	102	54.9	7.9	47	84.1	125.4	30.2
**pH**	80	6.8	2.3	0.5	111	6.9	2.2	0.4	51	6.7	4.7	0.8

**Table:** (**b**)

Microbiological/Physicochemical Parameter	Toilet Soil				Toilet Drainage Soil				Household Garden Soil			
	*n*	$\bar{x}$	R	SD	*n*	$\bar{x}$	R	SD	*n*	$\bar{x}$	R	SD
**Coliforms (CFU/100 mL)**	42	1832.6	2397	946.1	117	1944.7	2397	862.9	87	2023.9	2397	819.6
***E. coli* (CFU/100 mL)**	42	1579.2	2397	1032.2	117	1647.6	2397	997.9	86	1673.2	2397	1011.2
**Phosphate (mg/L)**	40	1.5	12.5	2.21	114	2.7	45	6.3	86	1.7	12.1	2.2
**Nitrate (mg/L)**	40	1.0	26.4	4.2	114	1.5	32.5	4.6	87	0.3	7.2	1.0
**Ammonia (mg/L)**	41	1.9	50	8.5	116	2.2	50	9.1	86	0.5	22	2.5
**Electrical Conductivity (uS/cm)**	41	196.9	1949.3	342.3	114	123.8	1172.3	178.2	88	97.8	1682.9	218.4
**Temperature (°C)**	40	26.3	9.2	2.1	114	26.3	9.8	2.1	88	26.4	10	2.1
**Dissolved Oxygen (mg/L)**	39	85.2	97.1	25.1	108	83.4	104.3	27.4	84	83.8	104.7	29.5
**pH**	40	6.6	4.0	0.9	114	6.6	4.7	0.9	88	6.6	4.1	0.7

**Table A4:** Variables used in Exploratory Factor Analysis. SHW = source of drinking water; TDS = toilet drainage soil; DO = dissolved oxygen; CFU = colony forming units.

Variable
1. Nearest road (m)
2. Elevation (m)
3. Drainage (0–4)
4. Substrate (0–4)
5. House condition (0–4)
6. Excreta disposal (0–4)
7. Garden position (0–4)
8. Bathing environs (0–4)
9. Drinking water environs (0–4)
10. Drinking water storage (0–4)
11. Housing density (0–4)
12. Solid Waste (0–4)
13. Toilet smell (0–4)
14. Coliforms_SHW (CFU/100 mL)
15. E. coli_SHW (CFU/100 mL)
16. Phosphate_SHW (mg/L)
17. Nitrate_SHW (mg/L)
18. Ammonia_SHW (mg/L)
19. Turbidity_SHW (FTU)
20. Temperature_SHW (oC)
21. Conductivity_SHW (μS)
22. DO_SHW (mg/L)
23. pH_SHW
24. Coliforms_TDS (CFU/100 mL)
25. E. coli_TDS (CFU/100 mL)
26. Phosphate_TDS (mg/L)
27. Nitrate_TDS (mg/L)
28. Ammonia_TDS (mg/L)
29. Temperature_TDS (oC)
30. Conductivity_TDS (μS)
31. DO_TDS (mg/L)
32. pH_TDS
